# Selected trace elements and metals in groundwater within Permian sediments near Olkusz (Zn-Pb ore mining region, S Poland)

**DOI:** 10.1007/s11356-018-2953-7

**Published:** 2018-09-10

**Authors:** Adam Postawa, Jacek Motyka

**Affiliations:** AGH University of Science and Technology, Al. Mickiewicza 30, 30-059 Krakow, Poland

**Keywords:** Neutral mining drainage, Zn-Pb ore mining, Heavy metals, Toxic elements, Olkusz region

## Abstract

The extensive mining of Zn-Pb ores in the Olkusz region resulted in significant changes of both water table level and chemical composition of water in all aquifers in the area. This was caused by intensive dewatering of mining excavations and development of a thick aeration zone reaching 150 m in a central part of the area. That created favorable conditions for oxidation of metal sulfides occurring in the ore-bearing dolomites (Middle Triassic) and started the process of forming readily soluble hydroxysulphates which then migrated to lower aquifers, including the Permian. As a result of those processes, various metals and other elements toxic to the water environment appeared in leaks observed in mine galleries. Changes in concentrations of selected elements (Fe, Mn, Zn, Pb, Cu, Ba, Ni, Co, As, Cr, Hg, Tl, Ag, Cd, B) in mine waters over the period of the last nearly 50 years were described. Water samples were collected from exploratory boreholes, piezometers, and wells located in investigated area inflows and seepages occurring in shafts and drifts excavated in Permian conglomerates. Mean concentrations of metals (Pb, Cd, Cr, Hg, Tl) and other toxic elements were surprisingly low; Pb, 3.94 μg/L; Cd, 0.2 μg/L; Cr, up to 2.26 μg/L; Hg, 0.25 μg/L; Tl, 3.59 μg/L; and As, 6.31 μg/L. However, the observed concentrations varied significantly over time, reaching respectively up to 190 μg Pb/L, 60 μg Cd/L, 15.6 μg Cr/L, 2.67 μg Hg/L, 81.3 μg Tl/L, and 155 μg As/L.

## Introduction

Underground mining of ore deposits usually leads to significant changes in surrounding environments. It affects land surface and both surface and groundwater. From a hydrogeological point of view, the most important is changes in water table levels and transformations of the chemical composition of groundwater. Depending on the type of rock hosting the ore and the type of ore minerals, the influence on water chemistry could vary significantly but usually leads to deterioration of water quality. The most common phenomenon associated with sulfide-type ores is the production of acidic solutions, which result from oxidation of various metals’ sulfides (e.g., pyrite, marcasite). The problem of acid mine drainage or acid rock drainage is widely described in literature (e.g., Banks et al. 1997; Gray 1997; Sainz et al. 2003, 2004 Johnson and Hallberg 2005; Grande et al. 2010, 2013, 2018; Bauerek et al. 2013), but some aspects of neutral or circumneutral drainage are still not fully recognized. This applies to ore deposits occurring in carbonate rocks and the presence of toxic metals and other microelements in mine waters. Problems induced by ore mining do not stop with mine closure but continuously affect the environment for many decades (Younger 1997; Cidu et al. 2001; Leblanc et al. 2000; Aykol et al. 2003; Cidu et al. 2005; Cidu et al. 2009; Frau et al. 2009; Sracek et al. 2011, 2012; Majzlan et al. 2018).

Sulfide ores of zinc and lead occur in numerous locations in the world. One of them is the Olkusz Zn-Pb ore mining region (Mississippi-Valley-type Zn-Pb ore region). Geologically, it is a part of the Cracow-Silesian monocline, a regional tectonic structure with the SE-NW direction formed as a result of the Alpine orogeny and located in southwestern Poland. It consists of Mesozoic, Triassic, Jurassic, and Cretaceous formations overlying discordantly on the fault bend fold Paleozoic structures formed during the Variscan orogeny.

Lead and zinc mining in the Olkusz region reaches back to neolith, which was confirmed by documents from the twelfth century. Initially, galena rich in silver was extracted from Triassic deposits located above the groundwater level. In the seventeenth century, a few adits were excavated to drain water from ore-bearing rocks and allow the exploitation to reach deeper. It resulted in depletion of the water table by 30 m in surrounding areas. This moment is considered the beginning of mining drainage in the Olkusz area. The most intensive mining drainage of the Triassic aquifer dates back to the second half of the twentieth century. It related to the development of the Bolesław mine and then establishing two new mines called: Olkusz and Pomorzany. Construction of the Pomorzany mine, which began in 1969, is especially important. The main accessing excavations of this mine were carried out in the Permian sediments (molasses) that underlie Triassic sediments. Starting from the early 1990s, another part of the Zn-Pb deposit was accessed by the Olkusz-sublevel mine and by galleries carried out in the Permian molasse.

Intensive mining drainage caused the development of a large regional cone of depression with an estimated surface area of about 350 km^2^ (Adamczyk 1990). In the central part of this area, the water table has dropped about 150 m (in the Triassic aquifer). The decrease of the water table was observed in all aquifers, including Quaternary, Jurassic, and Permian. That created a thick aeration zone and facilitated favorable conditions for oxidation of sulfate minerals occurring in abundance in Triassic carbonates. As a result of this process, large quantities of acidic solutions were produced, which were then buffered in contact with carbonate rocks (limestones and dolomites). A pH value of mine waters from leaks observed in the galleries carried out in Permian sediments has not changed significantly. It ranges from 6.4 to 8.4, at the initial stage of Pomorzany mine development in the early 1970s to 6.1–9.14 in 2015. However, important changes in the chemical type of water were observed. Within the Permian aquifer, the chemical type of the water has evolved significantly (Motyka and Czop 2006; Motyka and Postawa 2013).

Fluctuations of pH value and moreover changes in oxidation-reduction conditions facilitated mobility of Fe, Mn, and other red-ox sensitive elements. Some of them are well known as elements toxic to humans (e.g., Pb, Hg, Ni, As, Cd, Cr, Sb, Tl, or U) (Borgmann et al. 1998; Kazantzis 2000; Bagchi et al. 2002; WHO 2011; Riyaz et al. 2013; Belzile and Chen 2017; Lukaszewski et al. 2018; Pavoni et al. 2018). Some of the investigated elements are not considered severely toxic to humans, but they are still toxic to organisms living in a water environment (e.g., Zn, Cu, B, Ag, Co) (Hogstrand and Wood 1998; Marr et al. 1998; De Schamphelaere et al. 2007; Ebrahimpour et al. 2010; Brix et al. 2011).

The primary goal of this study was a characterization of the chemical composition of groundwater occurring within Permian sediments, especially in regard to metals and other trace elements. It was also an attempt to assess the influence of long-term mining activities carried out in the investigated area on the contents of these elements. It is an attempt to summarize and utilize the results of nearly 50 years of collecting unique hydro-chemical data.

## Materials and methods

### Geological settings

**Fig. 1:**
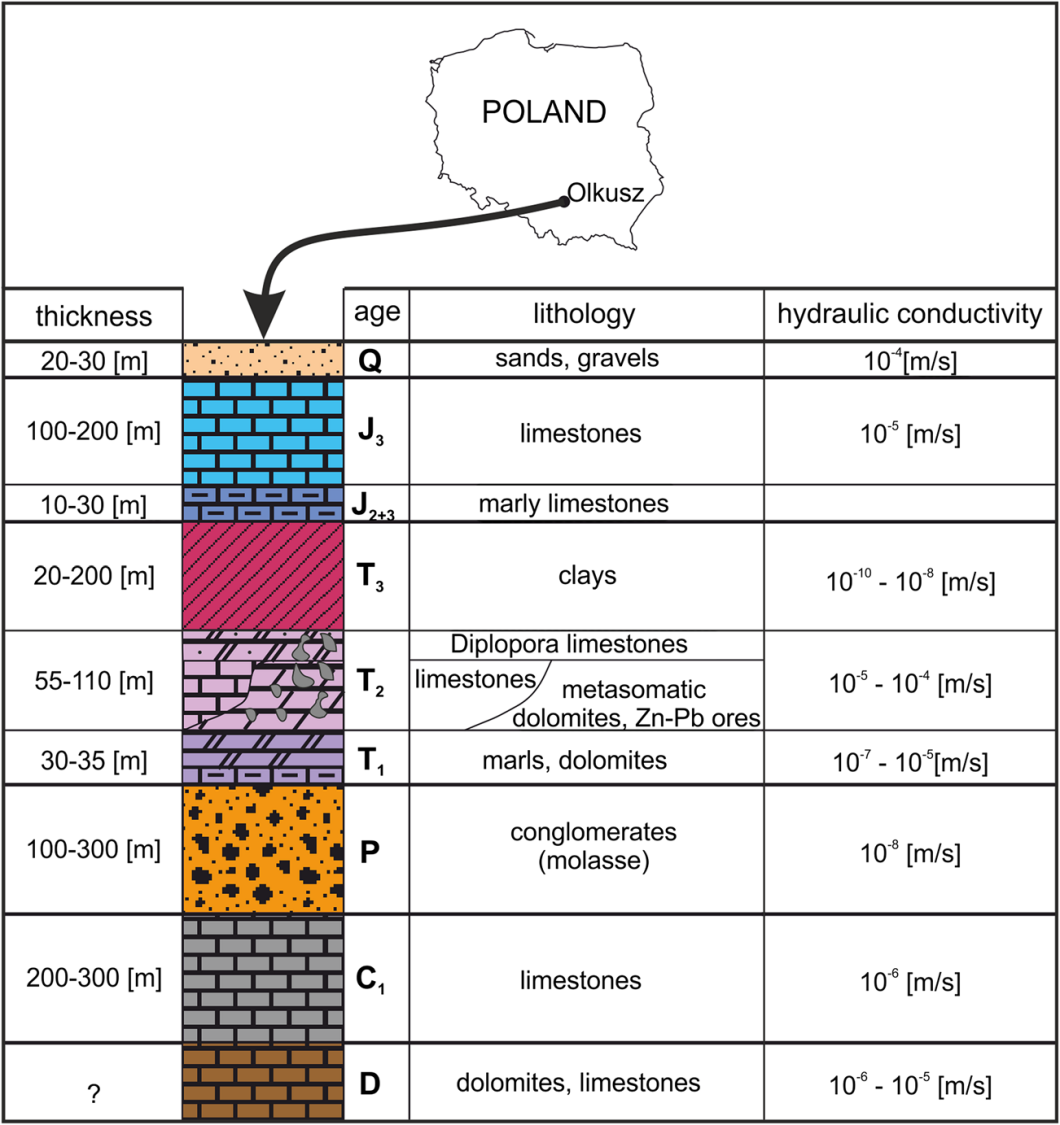
Synthetic geological profile of the Olkusz region

Figure 1 presents a synthetic geological profile from the Olkusz region. It also presents hydraulic conductivities (estimated ranges of orders of magnitudes) of main rock types occurring in the area.

During Permian, in the Olkusz region, Variscan tectonic movements formed a narrow but relatively deep mid-montane trench of the NW-SE direction between the elevated tectonic blocks of Upper Silesia and Lesser Poland (Buła 2000). This trench was covered with typical molassic formations, which consist mainly of conglomerates of fragments from older Paleozoic rocks, mostly limestones and Devonian and Carboniferous dolomites, as well as quartzites, porphyries, and melaphyres. The maximum thickness of the Permian sediments reaches approximately 300 m (Fig. 1).

Triassic rocks are located discordantly on different Paleozoic sediments. They are represented by the sediments of the Middle and Upper Triassic: Rhaetian, Mushelkalk, and Keuper. The Mushelkalk is composed of carbonate rocks, mainly limestones and dolomites, including also metasomatic ore-bearing dolomites, with Zn-Pb ore deposits. The primary ore minerals include sulfides of zinc (sphalerite) and lead (galena), accompanied by copious amounts of iron sulfides (i.e., pyrite and marcasite) (Sass-Gustkiewicz 1997; Mayer and Sass-Gustkiewicz 1998). In zones where the deposits are oxidized, calamines (gossans) occur. The thickness of shell limestone deposits reaches approximately 110 m (Fig. 1). The Upper Triassic deposits (Keuper) are formed in the terrestrial facies with interlayers of shallow sea facies deposits. Spotted clays, claystones, and shales prevail there, among which there are thin inserts of carbonate rocks and sandstones. The thickness of Upper Triassic rocks reaches approximately 200 m (Fig. 1).

The Jurassic sediments occur in the northern and eastern parts of the Olkusz region. They are represented by the Middle and Upper Jurassic rocks. The Middle Jurassic rocks include mostly marls and subordinately also clay-conglomerate rocks. The thickness of the Dogger rocks is small and usually falls within the range of 10–30 m. The Upper Jurassic (Malm) includes mostly different lithological variations of limestones with the marl layer in the bottom part. The thickness of the Malm deposits reaches approximately 200 m (Fig. 1).

Mesozoic and Paleozoic rocks are covered by Quaternary sediments. They are dominated by fluvioglacial sands with gravel, rubble, dust, silt, and clay inserts. On hilltops, built of older rocks, these are weathered clays (regolite) with a thickness of 1–2 m. The thickness of fluvioglacial deposits is usually within the range of 20–30 m (Fig. 1).

### Hydrogeological conditions

In the Olkusz region, there are five aquifers: Quaternary, Jurassic, Triassic, Carboniferous, and Devonian (Wilk and Motyka 1977).

A Quaternary aquifer is formed by sands with gravel inserts. The geometric mean value of the hydraulic conductivity for these sediments, determined based on the results of pumping tests, is equal to 2.5·10^−4^ m/s (Motyka and Wilk 1976). This aquifer is recharged by infiltrating atmospheric precipitation and by the inflow of water from other aquifers in areas of direct and indirect hydraulic contact. It is drained by surface watercourses and by the outflow of water to lower situated aquifers in the area influenced by mining drainage of the Olkusz Zn-Pb ore mines.

The Jurassic aquifer is built by platy, rocky, and chalky limestones of the Upper Jurassic. It is a fissure-karst type of aquifer. The geometric mean value of the hydraulic conductivity, determined during pumping tests, amounts to 1.6·10^−5^ m/s (Motyka and Wilk 1976). It is recharged by infiltrating atmospheric precipitation and drained by surface watercourses, intake wells, and outflow of water to other aquifers in areas of direct and indirect hydraulic contact (Wilk and Motyka 1977).

The Triassic aquifer is formed by dolomites and limestones of upper Bundsandstein and Mushelkalk. It is a pore-fissure-karst type aquifer (Motyka 1988; Motyka 1998). The geometric mean value of hydraulic conductivity of Triassic carbonates is equal to 6.5·10^−5^ m/s (Motyka and Wilk 1976). The dichotomy of permeability of these rocks is apparent, as the modal value of hydraulic conductivity for the shell limestone is approximately 9.0·10^−5^ m/s, while for the Bundsandstein sediments are approximately 7.0·10^−6^ m/s (Motyka and Wilk 1976). The discussed aquifer is supplied by infiltrating atmospheric precipitation in areas of its outcrop and by the inflow of water from other aquifers in areas of direct and indirect hydraulic contact. Infiltration of water from surface watercourses in areas covered by the drainage of the Olkusz Zn-Pb ore mines is an essential element of groundwater recharge. The Triassic aquifer is drained mainly by excavations of these mines, where currently about 5 m^3^/s of water flows in.

Carbonate Carboniferous and Devonian rocks form a common Paleozoic aquifer complex. It is a fissure-karst type aquifer. It is poorly investigated in terms of hydrogeological characteristics. The values of hydraulic conductivity, determined based on the results of pumping tests in two boreholes, were 7.6·10^−6^ m/s and 7.0·10^−5^ m/s respectively. Recharge and drainage of the Paleozoic aquifer occur in the areas of hydraulic contacts with other aquifers (Adamczyk et al. 1978).

Permian conglomerates are sometimes considered semipermeable sediments from a water supply standpoint (in terms of hydrogeological properties). The study on permeability, conducted in 14 boreholes, allowed determination of the hydraulic conductivity of these rocks. Values of these parameters fit within a wide range (i.e., from 1.0·10^−8^ to 1.6·10^−5^ m/s). Conglomerates have a higher permeability in the zones of fractures, associated with faults (Motyka and Postawa 2013).

**Fig. 2:**
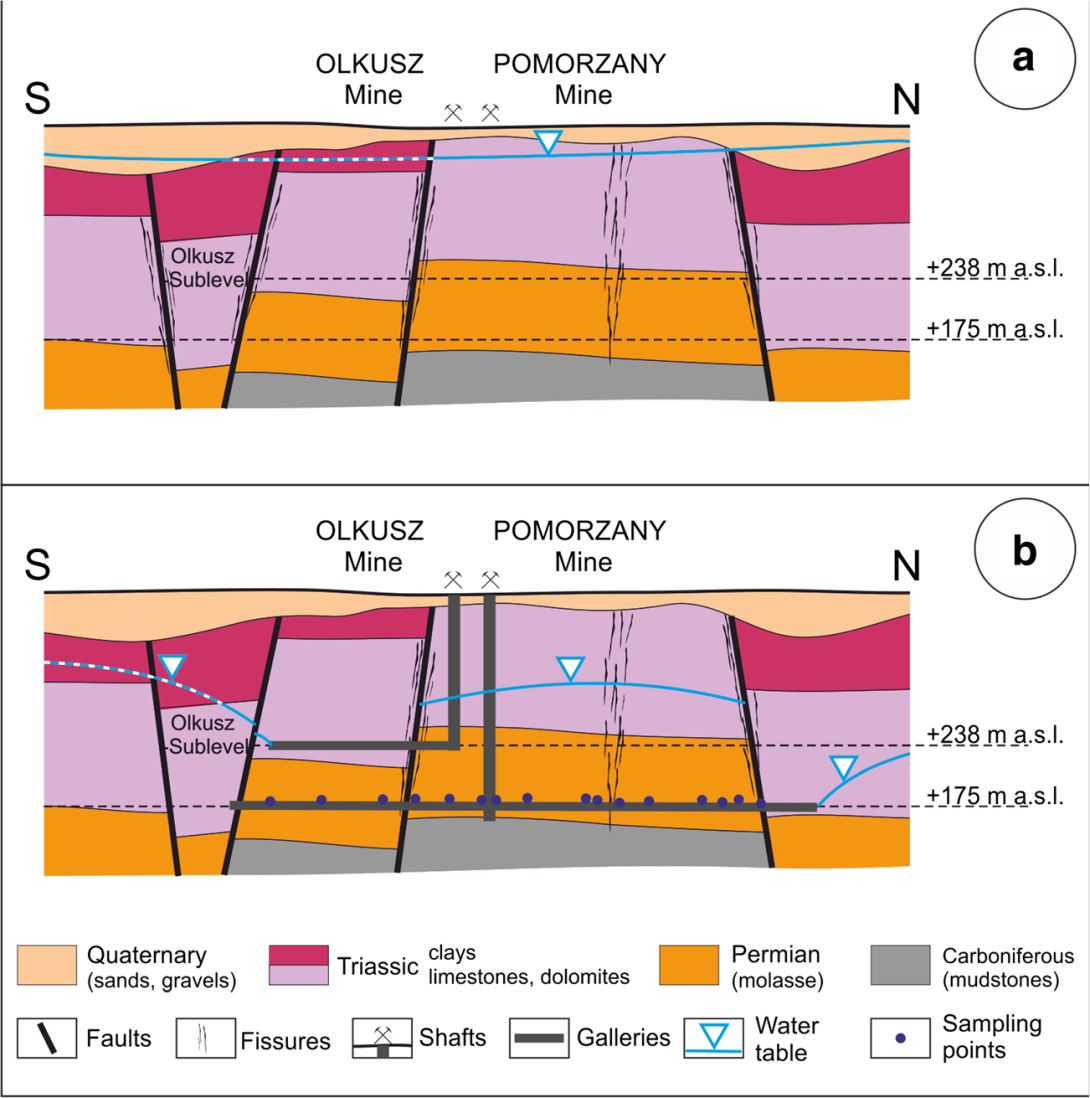
Schematic hydrogeological cross-section across the Olkusz region: **a** Natural conditions. **b** A period of intensive mining activities (around 1985)

The schematic hydrogeological cross-sections (Fig. 2) present the situation at the initial stage (prior to extensive mining) compared to the situation from around 1985. Significant changes in water table levels were caused by intensive dewatering of mining galleries.

### Mining context

In the second half of the twentieth century, there were three active mines of Zn-Pb ores in the Olkusz region (i.e., Bolesław, Olkusz, and Pomorzany). Mining excavations in the Permian conglomerates were performed in the Pomorzany mine at the level of about + 175 m a.s.l., due to the approved project of releasing Zn-Pb ore deposits, lying in the down-throw side of the Pomeranian fault, a regional dislocation in this area. According to the abovementioned project, the Pomorzany deposits started to be released in 1969 by excavations drilled from the Olkusz mine, whose main excavations were located at + 235 m a.s.l. in carbonate Triassic rocks. Therefore, completion of the project intended to release this deposit, which required a suitable system of excavations in the Permian conglomerates that would allow crossing the Pomorzany fault. The first excavations crossed the Pomorzany fault in September 1973.

Due to the depletion of Zn-Pb ore resources, the Bolesław mine was closed in 1996, and its excavations were spontaneously flooded. Submersible pumps were installed in the main shaft to intake industrial water. In 1999, the closure of the Olkusz mine began, but its dewatering was maintained due to the exploitation safety reasons of the Olkusz-sublevel Zn-Pb ore deposits lying about 60 m below the drain galleries of the closed down Olkusz mine.

Zinc and lead ores are currently exploited in the Olkusz region only in the Pomorzany mine and within the mentioned Olkusz-sublevel deposit.

### Sampling

**Fig. 3:**
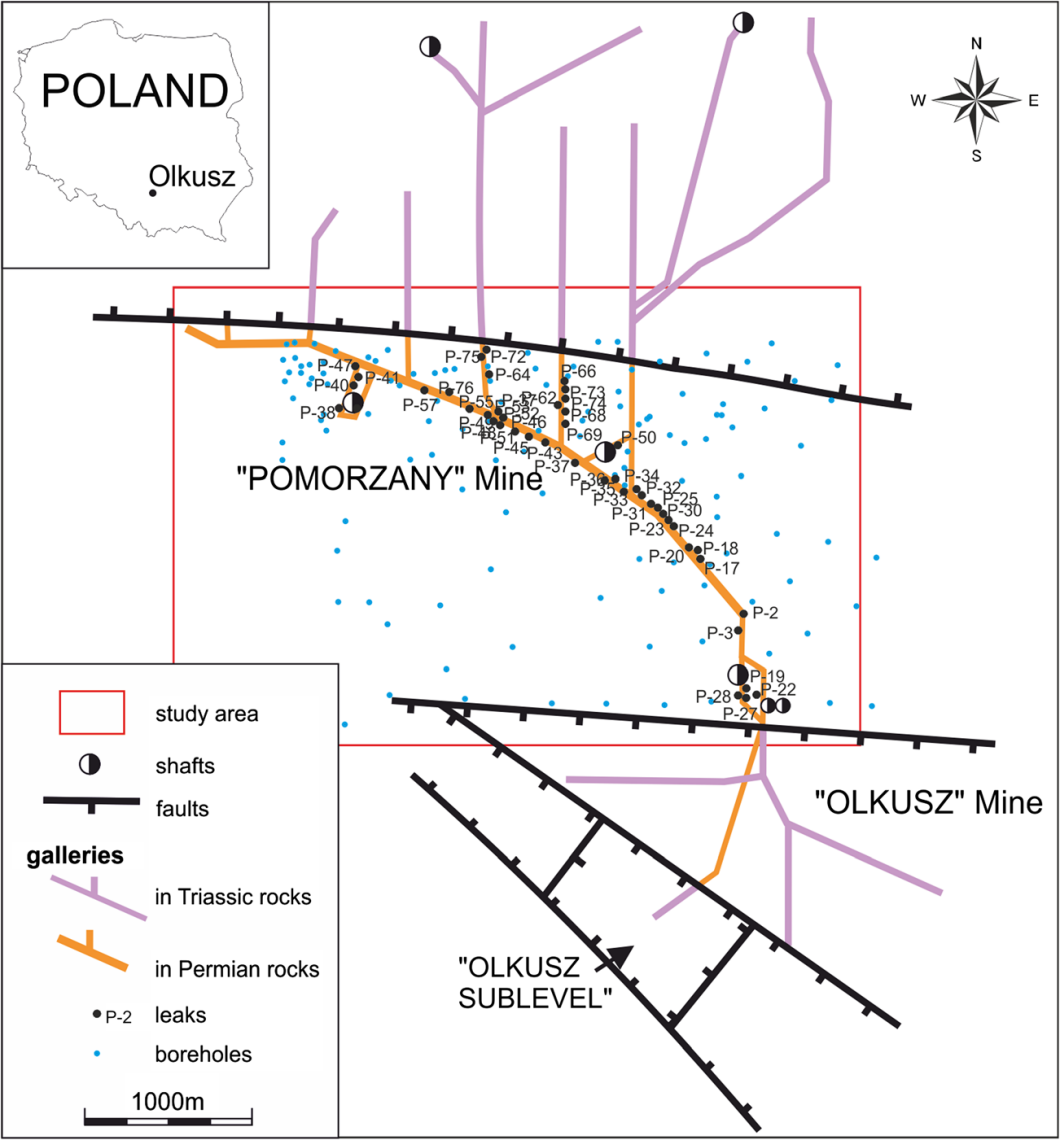
Location of sampling points

The samples of water from leaks from the Permian conglomerates released by the mining excavations of the “Pomorzany” mine have been collected since 1971 (i.e., since they appeared). Until 1976, the water samples were collected with the progress of mining works and implementing of the part of the project on releasing the Pomorzany deposits, which concerned mining excavations in the Permian conglomerates. After some time, some of the leaks disappeared, and only a few of them were permanent enough to allow collection of water samples for several years. Until now, from the total initial population of about 80 leaks, only 20 leaks are accessible, from which water samples were collected with variable frequency, usually every 2–3 years. During the period of 1980–1995, the samples of water were collected very rarely and only from some of the active leaks from the Permian conglomerates. For this reason, there is no continuity of sampling during this period. The last samples were collected in 2016. Locations of sampled leaks are presented on a map showing main galleries of Pomorzany and Olkusz mines (Fig. 3).

Samples were also collected from exploratory boreholes drilled during deposit documentation and exploration stages, piezometers, and wells located in the investigated area. In total, over 400 samples were collected during the investigation period (1967–2016). However, one should keep in mind that the numbers of samples and the numbers of determinations of particular ions/elements are not the same. Unfortunately, in many samples, only selected parameters were determined; thus, the number of useful results of microelements’ determinations is smaller and varies from 193 determinations for cadmium up to 255 for zinc.

All samples were collected as non-filtered samples and delivered to a laboratory within 24 h. Since 1990, directly upon delivery to the laboratory, samples were filtered using 0.45 μm filters, and part of each sample intended for metals determination was acidified with nitric acid to a pH < 2.

### Analytical methods

Chemical analyses of all samples were performed by a hydro-geochemical laboratory of Department of Hydrogeology and Engineering Geology, AGH University, Krakow. In 2009, the laboratory was accredited by the Polish Centre for Accreditation (accreditation number AB 1050). The laboratory has fully implemented an internal quality assurance and quality control system (QA/QC), which fulfills the requirements of the ISO/IEC 17025 standard. The QA/QC system is based on analyses of control samples (blanks, duplicates, and spiked samples) and certificated standard materials (ION-96.3, ION-96.64, and TMDA-4.3 by Environment and Climate Change Canada and 1643a and 1643e by the U.S. National Institute of Standards and Technology). The laboratory participates in proficiency-testing programs and interlaboratory comparisons (both national and international) achieving satisfying results.

Chloride concentrations were determined using the Mohr method (i.e., titration with silver nitrate, in accordance with ISO 9297 standard). Concentrations of hydrocarbonates were calculated on the basis of alkalinity determinations, which were performed by titration with hydrochloric acid. Sulfate concentrations, until 1990, were determined using the gravimetric method with drying of residue.

The total sulfur content was determined, and then sulfate concentration was calculated. In samples collected before 1990, calcium was determined by titration with ethylenediaminetetraacetic acid (EDTA). Magnesium concentrations were calculated on the basis of total hardness and calcium. Sodium and potassium concentrations were determined by flame emission photometry. Since 1990, all these metals have been determined using the inductively coupled plasma atomic emission spectrometry (ICP-AES) method.

Since 2001, concentrations of all microelements were determined using the inductively coupled plasma mass spectrometry (ICP-MS) method (ELAN 6100 spectrometer, PerkinElmer). This allowed for significant improvement in the quality of metals’ determinations in terms of practical detection limits.

## Results and discussion

**Fig. 4:**
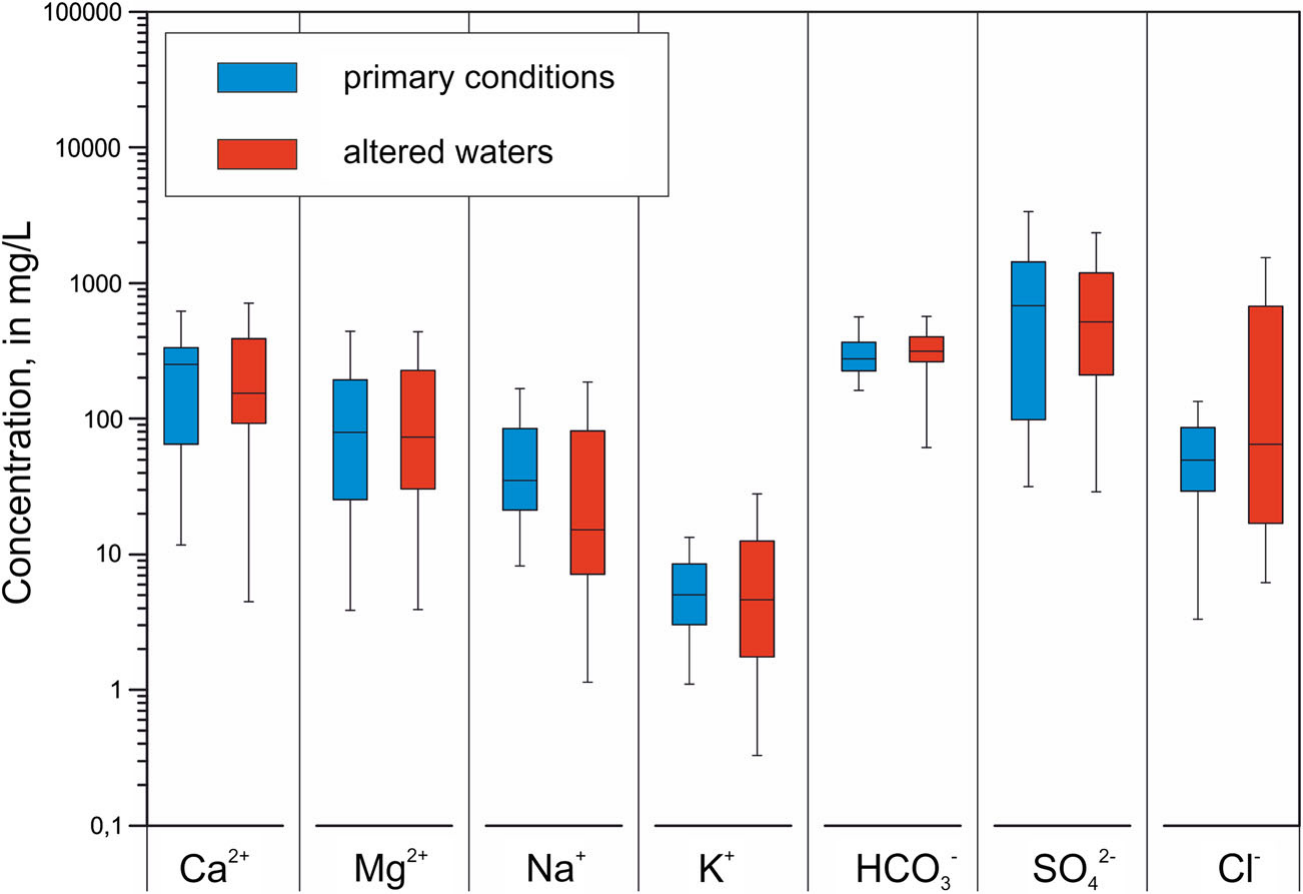
Concentrations of major ions in groundwater in Permian aquifer

Samples collected from the leaks during the period of drilling mining excavations in the Permian conglomerates presented very diversified chemical compositions. Total dissolved solids (TDS) of these waters ranged from 0.33 to 21.6 g/L, whereas its dependence on the depth of the leak with respect to the Permian conglomerate roof was evident (Motyka and Postawa 2013). In waters with the lowest TDS, Ca, Mg, and HCO_3_ ions dominated; while with increasing TDS, the share of Na, SO_4,_ and Cl ions increased. Therefore, the hydro-chemical type of water has changed from Ca-Mg-HCO_3_ for waters with TDS of 0.5 g/L through multi-ion waters with different combinations of the main ions to the Na-Cl type for waters with TDS value of 10–20 g/L. It was accompanied by an increase in average concentrations of all major ions except for potassium (Fig. 4).

With the progress of mining works, the TDS of leaks observed in earlier-excavated galleries decreased very quickly due to the inflow of fresh water from the upper part of the Permian conglomerates and the overlying Triassic deposits. The share of calcium and magnesium ions in the chemical composition of water from those leaks increased significantly.

After 1985, when a new quasi-fixed hydrodynamic balance in conditions of mining drainage of the Permian conglomerates was established, most leaks ceased to exist. Only those from the western part of the mine excavations remained active, which were recharged mainly with water from the overlying ore-bearing Triassic deposits. Readily soluble hydroxysulphates of calcium (gypsum), magnesium (epsomite, hexahydrite), and iron (melanterite) were formed because of weathering sulfide minerals (mostly marcasite) occurring in these rocks. The inflow of water with high TDS from the Triassic aquifer changed the original character of leakage waters from the Permian deposits. It could be observed that TDS values significantly increased to a few or several grams per liter, while the hydro-chemical type had changed from Ca-Mg-HCO_3_ to Ca-Mg-SO_4_ and Mg-Ca-SO_4_ to Mg-SO_4_. It is a typical phenomenon, commonly occurring in such conditions (Hermann and Neumann-Mahlkau 1985; Lee et al. 2001; Cidu et al. 2007; Hidalgo et al. 2010).

By the mid-1980s, the concentration of iron, manganese, zinc, and lead only had been analyzed in a few samples. The iron concentration ranged from 0.04 to 2.6 mg/L (mean of 0.36 mg/L), manganese from 0.02 to 0.07 mg/L (mean of 0.025 mg/L), zinc from 0.13 to 5.7 mg/L (mean of 0.31 mg/L), and lead from 0.06 to 0.19 mg/L (mean of 0.08 mg/L). It should be emphasized here that different analytical methods used at that time provided inconsistent results and allowed detection of an element only when its concentration in the aqueous solution was significantly higher compared to that with the currently used, more advanced, analytical methods.

Starting from 1990, the use of new analytical methods, ICP-AES and ICP-MS (since 2001), allowed for a considerable increase in the number of analyzed microelements and to reach the detection level of parts of micrograms per liter. Samples collected from leaks in the period of 2001–2016 (241 samples) retained almost unchanged chemical composition, similar to those recorded in the 1980s. That occurred, probably, after the stabilization of the new hydrodynamic balance caused by mine drainage. The concentration of iron in these samples ranged from 0.007 to 40.1 mg/L, manganese from 0.0009 to 3.96 mg/L, zinc from 0.013 to 69.8 mg/L, and lead from 0.00008 to 0.135 mg/L (see Table 1).

**Table 1:** Concentrations of selected elements in water within Permian sediments near Olkusz. Summary statistics

	1967–1985					1985–2016					98/83/EC parametric value
	N	AVG	Median	Max	SD	N	AVG	Median	Max	SD	
Element	–	mg/L				–	mg/L				mg/L
Ag	0	–	–	–	–	241	0.127	0.0002	2.05	0.376	–
Al	2	0.022	0.022	0.04	0.025	214	0.0098	0.0004	0.202	0.0282	0.2
As	1	0.01	0.01	0.01	–	204	0.008	0.0036	0.155	0.015	0.01
B	3	1.75	1.78	1.91	0.45	204	0.069	0.034	0.388	0.085	1.0
Ba	0	–	–	–	–	241	0.378	0.027	12.600	1.22	–
Br	23	9.47	1.61	76.7	21.6	204	0.063	0.027	0.581	0.088	0.01
Cd	3	0.0273	0.017	0.06	0.0289	190	0.0092	0.0002	0.6	0.0316	0.05
Cr	0	–	–	–	–	204	0.001	0.0022	0.0156	0.002	0.05
Fe	8	3.04	0.355	2.6	1.870	237	2.44	0.459	40.69	5.23	0.2
Hg	0	–	–	–	–	204	0.00042	0.00025	0.00267	0.00083	0.001
Mn	3	0.037	0.02	0.07	0.029	241	0.398	0.153	3.963	0.569	0.05
Ni	0	–	–	–	–	205	0.0425	0.027	0.372	0.095	0.02
Pb	9	0.106	0.08	0.19	0.052	204	0.0055	0.0039	0.19	0.0128	0.01
Se	1	1.05	1.05	1.05	–	204	0.008	0.003	0.107	0.015	0.01
Tl	0	–	–	–	–	204	0.006	0.0036	0.081	0.01	–
Zn	11	5.002	0.31	51.7	15.4	244	4.25	1.24	69.8	7.52	–

*N*, number of determinations; *AVG*, arithmetic mean; *SD*, standard deviation.

Lack of reliable data covering the whole investigated period does not allow clear determination of how the concentrations of microelements have changed. Only the past 20 years of water leaks from the Permian deposits could be included in this study. However, in 2001 and 2007, samples collected from three leaks showed chemical composition practically identical to the composition observed before 1985. It can be assumed that the results of analyses of water from the abovementioned three leaks can be used as a reference level. The concentrations of Fe, Mn, Zn, Pb, Ni, Co, Tl, Cd, and Al in waters of the entire examined population of leaks are significantly higher than in water not affected by chemical transformation. Similar concentrations in both separated collections of water were found for Ba, As, Cr, and Sb.

Summary statistics for all sampled points are presented in Table 1. The results are divided into two groups: a group representing samples collected prior to 1985 representing “primary conditions” and a group representing samples collected after 1995 (i.e., with the chemical composition significantly altered by mining activities).

**Fig. 5:**
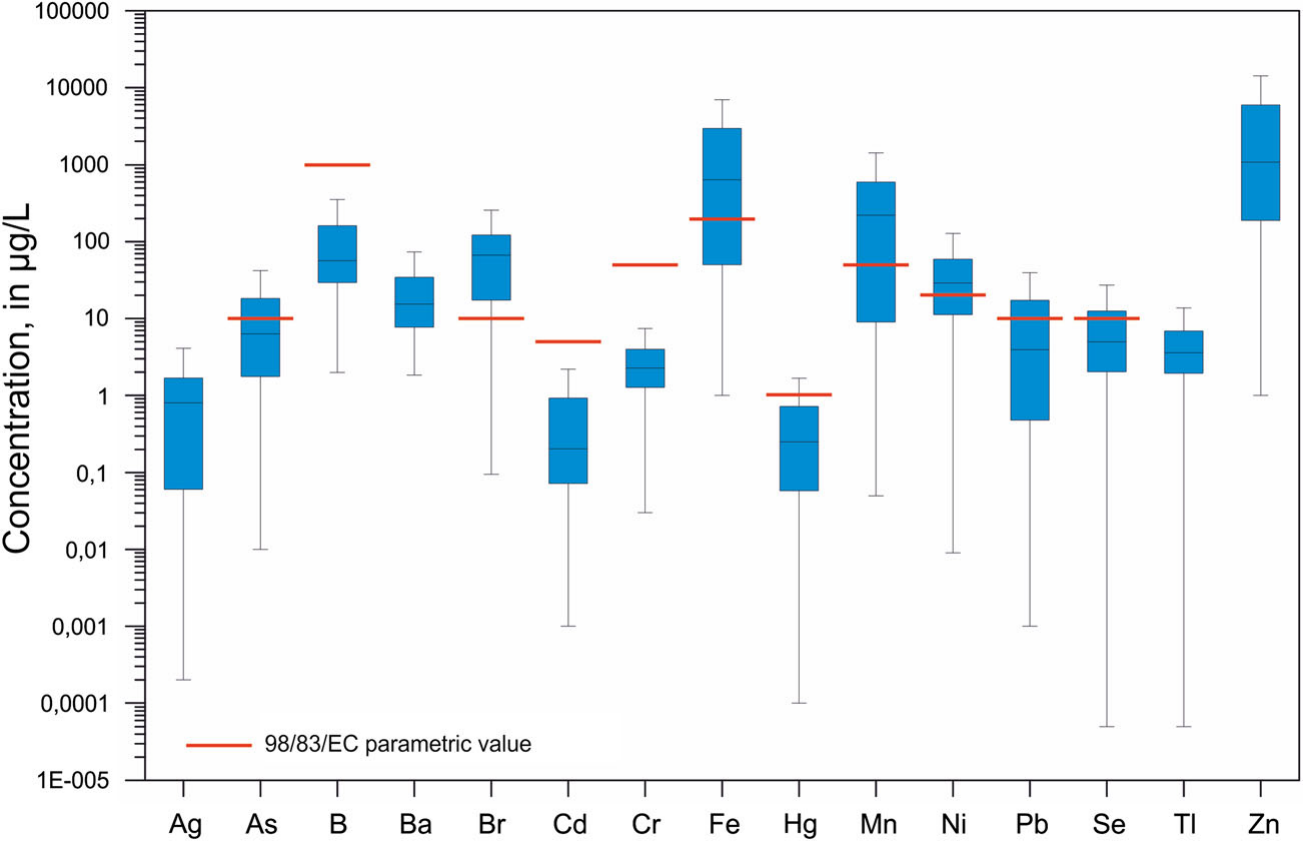
Concentrations of selected elements in water samples from mine leaks in the Olkusz region (box-whiskers plot without outliers)

Average concentrations of most studied microelements do not exceed parametric values established for drinking water (98/83/EC 1998) and guideline values recommended by the WHO (2011), as presented in Fig. 5. The only exceptions are bromates, iron, manganese, and nickel. The mean concentration of iron in the whole studied period (1967–2016) was 2.7 mg/L, manganese 0.39 mg/L, and nickel 42.5 μg/L (however, concentrations of nickel were analyzed since 2001). The concentrations of lead, arsenic, and selenium only occasionally exceeded the parametric values (98/83/EC 1998). It is not possible to identify clear links between the degree of groundwater transformation caused by mining activity and concentrations of these elements. Elevated concentrations of iron manganese, nickel, arsenic, and other metals in both groundwater and surface water in the areas of Zn-Pb ore mining are reported in numerous studies (e.g., Cidu et al. 2001; Aykol et al. 2003; Sainz et al. 2004; Lee et al. 2005; Hidalgo et al. 2010; Sracek et al. 2012; Bauerek et al. 2013).

The highest concentration of all metals reached was zinc. Concentrations of this metal varied from single micrograms per liter to 69.8 mg/L. A high concentration of Zn in water is expected in existing conditions, as it was described by Cidu and others (Cidu et al. 2005).

However, the most concerning from a toxicological point of view are relatively high concentrations of thallium, which is well-known as a highly toxic element (Borgmann et al. 1998; Kazantzis 2000; Xiao et al. 2012; Belzile and Chen 2017). Concentrations of thallium in water from the Permian aquifer reached 81 μg/L. The parametric value in drinking water is not regulated in most countries, including EU. In the U.S.A., the Environmental Protection Agency has set a maximum contaminant level (MCL) in drinking water at 2.0 μg/L (with a maximum contaminant level goal, MCLG, of 0.5 μg/L).

The evolution of chemical composition of water from leaks from the Permian conglomerates in the initial phase of the mining drainage (transient conditions) consisted of reducing their TDS value because of freshwater inflow from the overlying Triassic aquifer (Motyka and Postawa 2013). The new hydrodynamic balance in the surroundings of mining excavations was stabilized approximately around 1985. This is confirmed by the results of monitoring of changes in the concentration of Cl ions in the selected leaks from the Permian deposits.

Weathering of sulfide minerals, which occurred in the ore-bearing dolomites (Middle Triassic), affected by the mining drainage, initiated the process of producing readily soluble hydroxysulphates (mainly of calcium, magnesium, and iron). Water from the Triassic aquifer, containing sulfates, calcium, and magnesium in high concentration, infiltrated the underlying Permian conglomerates (Motyka and Czop 2006; Motyka and Postawa 2013). Infiltrating water contained elements present in sulfides of iron, zinc, and lead as well. The concentration of the latter depended on their mobility and solubility of secondary minerals of these microelements in the geochemical environment reach in carbonate rocks (Hermann and Neumann-Mahlkau 1985; Hidalgo et al. 2010; Pavoni et al. 2018). Therefore, the highest concentrations were found for zinc (up to 69.8 mg/L) and iron (up to 40.7 mg/L). The process that inhibits the passage of iron to the aqueous solution is the precipitation of hydro-oxides of this element on the sidewalls of mining excavations. There were high concentrations of manganese, reaching up to 3.9 mg/L, found in leaks with a high proportion of water transformed by the above-discussed geochemical processes.

Because of the oxidation process of iron, zinc, and lead sulfides, there are abnormally high concentrations of some microelements in the transformed aqueous solutions. There are elevated contents of nickel, cobalt, and arsenic in the iron sulfides (marcasite, pyrite). The concentrations of these microelements in waters with transformed chemical composition reached 0.372 mg Ni/L, 0.015 mg Co/L, and 0.155 mg As/L. Zinc sulfides (sphalerite, wurtzite) are reached in cadmium (Mayer and Sass-Gustkiewicz 1998), thus, are the most probable source of cadmium in groundwater (Cidu et al. 2007). The concentration of Cd in transformed waters reaches 0.26 mg/L. Since the Middle Ages, galena was also used for the recovery of silver during metallurgical processing of lead ore. Galena, which contains increased levels of silver and thallium, could be a source of these elements in groundwater (Pavoni et al. 2018).

In the altered waters, compared to the natural ones (not affected by mining activities), elevated aluminum concentrations were also observed, reaching 0.4 mg/L. Most probably, this element passes into the solution because of buffering acidic solutions resulting from oxidation of metal sulfides on aluminosilicates, mainly on clay minerals. Such minerals occur commonly in the Triassic carbonate rocks as karst fillings and/or as the marl or clay inserts.

Natural waters contain significantly higher amounts of boron than the transformed ones. In natural water, the concentration of this element falls within the range of 1.5 to 1.9 mg/L while in transformed waters, it is up to 0.388 mg/L. Boron is an element present in elevated concentrations in natural waters associated with evaporates. Therefore, its higher concentration in waters dominated by chloride ion is justified. Waters infiltrating from overlying Triassic carbonate rocks have a significant share in transformed water and initially present low TDS values, without characteristics of water associated with evaporates, so presumably, the concentrations of boron in these waters were at low levels (i.e., of parts of mg/L).

## Conclusions

At the initial stage of mining drainage, groundwater occurring in Permian molasse demonstrated the distinct natural, chemical zonation. With an increase in the depth, counted from the ceiling of these sediments, concentrations of SO_4_ and Cl ions increased, with a decreasing share of HCO_3_. Because of mining drainage, a significant decrease in TDS values of water occurred in a relatively short period of time. A long-term effect was the production of readily soluble hydroxysulphates of calcium, magnesium, and iron. It was a result of natural geochemical processes (i.e., oxidation of metal sulfides [mostly pyrite, marcasite, galena]). In waters from leaks that were active for over 40 years, a significant change in TDS value and the chemical type took place. It has changed from initial Ca-Mg-HCO_3_ to Ca-Mg-SO_4_ and, in extreme cases, to Mg-SO_4_.

Oxidation of sulfides generated large portions of acid solutions, thus creating favorable conditions for production of relatively mobile metal ions. However, in an environment of carbonate rocks, these solutions were buffered, and the pH value of water has not been changed significantly, but concentrations of some metals and other microelements have.

In waters flowing into mining works excavated in the Permian sediments, maximum concentrations of Zn increased to 69.8 mg/L, Fe to about 40.6 mg/L, and Mn to about 3.9 mg/L. An increase in the concentration of toxic elements was also observed, especially Ni (up to 372 μg/L), As (up to 155 μg/L), Pb (up to 190 μg/L) of Cd (to 60 μg/L), and Tl (to 81 μg/L). The most severe concerns cause the presence of thallium, which is highly toxic and should be considered as an emerging pollutant associated with ore mining and processing. This requires future investigation.

Mine drainage and induced changes in the chemical composition of groundwater should always be considered when assessing environmental impacts of mining activities. Neutral mine drainage has less dramatic consequences than acid drainage, but still, it may cause an increase in concentrations of toxic elements in groundwater. Presently, contamination of groundwater within Permian sediments does not present a significant threat to the environment due to relatively low permeability (compared to Triassic limestones and dolomites), thus the negligible role of this aquifer in general groundwater flow.
